# Wolf-in-the-Tail

**Published:** 1898-06

**Authors:** 


### WOLF-IN-THE-TAIL.

EDITOR OF FARMERS' REVIEW: Each recurring spring there are in this vicinity a number of milch-cows that die soon after calving. The first impulse of the frightened owner is to send for a "doctor." We always have a good crop of "cow-doctors," in season and out. We make haste to secure the quickest one obtainable, and generally find him loafing around the village depot waiting for something to turn up. He is well equipped for his profession, as he understands it, and he is also the owner of a low-wheeled wagon suitable for a cow-hearse, and is familiar with the shortest route to the bovine potter's field. He visits the unfortunate cow, and after an apparently critical examination, he pronounces the disease as "wolf-in-the-tail." He don't show you the wolf, but assures you that it is there. Some of them—the alleged doctors—call it "worm-in-the-tail," and it is for that reason, I presume, they dose the cow with whiskey, probably upon the theory that in its distillation whiskey comes from the worm—and, therefore, naturally will "go for the worm." In most cases the cow dies, and you pay the whole bill to the one person, as he acts as both medical adviser and undertaker. Once in a while a cow has had but a light attack, and by the time the torturer has arrived has regained enough stamina to overcome the disease and his treatment, and lives, and for generations thereafter one hears of how Sam Johnson cured Jones's cow of "wolf-in-the-tail."

Your valuable paper is always ready to shed the light of progressive research upon the musty ignorance of the past, and I know of no more humane action that could be taken than to have your veterinary editor take up this subject and treat it freely. This is not a case where "ignorance is bliss," but where ignorance is damnable.

W. C. EGAN,
Victim.

HIGHLAND PARK, ILL.

There was a time, years ago, when ignorant bigots burned alleged witches at the stake; when farmers regulated their work according to the signs of the zodiac; when people, plethoric or poverty-stricken in blood, were, with equal copiousness, bled each spring by the village barber. Barbarous indeed were these practical evidences of densest ignorance, but happily they have largely disappeared before the bright light of education and humanity. But some remain, it seems, for, in the foregoing letter, we learn that there are actually people living in this age of education who believe in the myth termed "wolf-in-the-tail"—a relic of the days alluded to and akin in absurdity to the other cruelties and customs mentioned.

The man who believes in "wolf-in-the-tail" condemns himself out of his own mouth as an ignoramus capable of witch-burning, and anyone whose cow he has maltreated by the administration of vile nostrums calculated to clear the caudal appendage of its mysterious parasitic pest, has capital cause to commence a suit for damages or prosecute the offender for cruelty to animals. Strange as it may seem, the progressive State of Illinois refuses to enact a law preventing "wolf-in-the-tail" doctors and similarly qualified quacks from practising their inhuman, destructive profession (?), but our correspondent and other "victims" still have recourse to the Prevention of Cruelty to Animals Society, and should avail themselves of it.

It would seem unnecessary to state in these columns that there is not and never was any such disease as wolf-in-the-tail; that no educated man who knows anything about cattle believes in its existence, or that anyone so ignorant as to believe in it or its first cousin called "hollow-horn," is qualified to prescribe or administer drugs to animals.

Unquestionably the disease our correspondent speaks of as prevailing each spring among newly-calved cows is parturient apoplexy, commonly termed "milk-fever," and the prevalence of the trouble proves conclusively (1) that finely-bred cattle are kept; (2) that they are pampered in warm barns, and (3) that they are overfed upon rich foods. The disease is particularly liable to attack finely-bred cows, such as Jerseys; almost invariably chooses the "deep milkers" as its victims; is unknown among heifers and, as a rule, afflicts only cows having had three calves. It is a fearfully fatal disease, although a new method of treatment has enabled qualified practitioners to save many patients by aiding nature in her attempts at resuscitation so often rendered futile by ill-directed interference and drugs. It is peculiarly a disease of the plethoric animal, and follows usually an easy labor and prompt expulsion of the afterbirth. Recovered animals are liable to suffer from another attack with their next calf, and in such case almost invariably succumb.

To cure the disease—or, more correctly speaking, assist nature toward a recovery, for nobody but a quack would claim that he personally cured anything—requires the most intelligent application of common-sense ideas based upon practical experience, and the exhibition of drugs indicated by each symptom as it appears, together with properly directed nursing and observation of the laws of hygiene.

Happily, however, the disease may be prevented with almost absolute certainty if a few simple rules are correctly followed. Nature did not intend a cow to live without other exercise than mastication and digestion. Exercise, therefore, is necessary to conserve the health of both the pregnant cow and her fœtus. Inaction and warm atmosphere, together with highly nitrogenous and nutritious foods, beget plethora of the blood, obesity, and constipation. Knowing, then, that pure-bred cows of the dairy breeds having had three calves are hereditarily susceptible to "milk-fever," the aim of the owner and feeder should be to counteract natural tendencies and artificial conditions by treatment based upon nature's methods. Stated as concisely as possible, then, preventive treatment consists in "drying the cow off" at least six weeks prior to parturition, allowing exercise daily in a yard out of doors, easing the udder of a portion of the milk twice daily, if plentiful, at any time prior to the advent of the calf, and for a period of six weeks before that time cutting down the rations to a simple diet of sound hay and bran-mashes containing sufficient oil-meal to keep the bowels freely open. The last point is the most important, as it means prevention of plethora and constipation, and it will be found that as the tendency to the latter increases a few days prior to parturition it will be necessary to increase the daily allowance of oil-meal; at first merely mix a handful of pure oil-meal in each regular feed, but gradually increase the amount until the cow gets about twice or thrice as much the day or day before calving. Where oil-meal alone does not move the bowels sufficiently, add an ounce or two of epsom salts dissolved in warm water, and increase the amount just before calving. As soon as the calf comes, and before the afterbirth is expelled, give her a warm mash, very thin, nearly as thin as gruel, made as follows: 4 quarts coarse bran; $\frac{1}{2}$ pint linseed-meal; 1 tablespoonful salt; 1 tablespoonful tincture of arnica, and warm water to suit. If the cow does not clean readily, give her from 4 to 8 qnarts of whole oats dry, and if afterward there are premonitory symptoms of "milk-fever," such as restlessness, stepping up and down with hind feet, unsteadiness of gait, etc., give rectal injections of soapy warm water, and twenty drops of tincture of aconite, and repeat aconite in two hours, adding a stimulant.—A. S. ALEXANDER, M.D.C., V.S., Evanston, Ill., in *The Farmers' Review*, Chicago, March 2, 1898.

---

The *Breeders' Gazette*, Chicago, Ill., asks, for the benefit of cattle-growers, the following questions relative to a type of diarrhœa or scours which has carried off thousands of young calves:

1. Is it a new disease? State how long you have had experience with this fatal form of diarrhœa.
2. What in your opinion is the cause of this disease?
3. Describe the symptoms of the disease. (*b*) At what age does it appear? (*c*) How long do calves live? (*d*) What percentage recover?
4. Give particulars as to foods used and method of feeding the dams of calves that have succumbed to the disease.
5. Does it equally affect the calves of stabled cows and cows exercised daily in winter?
6. Does it equally affect suckling calves and hand-fed calves?
7. Describe the methods of treatment and medicines (*a*) that have failed, and (*b*) that have proved beneficial.

The Prussian Government has taken another step against the importation of our meat-products. Provincial authorities are directed to pay especial attention to the inspection of pork and other meats from this country.

It is interesting to observe the weekly increased number of advertisements of noted stock-breeders offering tuberculin-tested stock. This will very soon solve this important question among our breeders and save thousands of dollars annual loss.

